# Diet, obesity, and the gut microbiome as determinants modulating metabolic outcomes in a non-human primate model

**DOI:** 10.1186/s40168-021-01069-y

**Published:** 2021-05-05

**Authors:** Tiffany M. Newman, Carol A. Shively, Thomas C. Register, Susan E. Appt, Hariom Yadav, Rita R. Colwell, Brian Fanelli, Manoj Dadlani, Karlis Graubics, Uyen Thao Nguyen, Sivapriya Ramamoorthy, Beth Uberseder, Kenysha Y. J. Clear, Adam S. Wilson, Kimberly D. Reeves, Mark C. Chappell, Janet A. Tooze, Katherine L. Cook

**Affiliations:** 1 Department of Cancer Biology, Wake Forest School of Medicine, Winston-Salem, NC 27157 USA; 2 Department of Pathology, Section of Comparative Medicine, Wake Forest School of Medicine, Winston-Salem, NC 27157 USA; 3 Department of Neurosurgery and Brain Repair, USF Center for Microbiome Research University of South Florida Morsani College of Medicine, Tampa, FL USA; 4 Department of Internal Medicine-Molecular Medicine, Wake Forest School of Medicine, Winston-Salem, NC 27157 USA; 5 CosmosID, Rockville, MD USA; 6 Metabolon, Raleigh, NC USA; 7 Department of Surgery, Wake Forest School of Medicine, Winston-Salem, NC 27157 USA; 8 Comprehensive Cancer Center, Wake Forest School of Medicine, Winston-Salem, NC 27157 USA; 9 Department of Biostatistics and Data Science, Wake Forest School of Medicine, Winston-Salem, NC 27157 USA; 10 Wake Forest School of Medicine, 575 N. Patterson Ave, Suite 340, Winston-Salem, NC 27101 USA

**Keywords:** Metagenomic sequencing, Metabolomics, Western and Mediterranean diet, Body fat composition, *Prevotella copri*, *Eubacterium siraeum*, Urinary carnitine metabolites, Uremic toxins

## Abstract

**Abstract:**

**Background:**

The objective of this study was to increase understanding of the complex interactions between diet, obesity, and the gut microbiome of adult female non-human primates (NHPs). Subjects consumed either a Western (*n*=15) or Mediterranean (*n*=14) diet designed to represent human dietary patterns for 31 months. Body composition was determined using CT, fecal samples were collected, and shotgun metagenomic sequencing was performed. Gut microbiome results were grouped by diet and adiposity.

**Results:**

Diet was the main contributor to gut microbiome bacterial diversity. Adiposity within each diet was associated with subtle shifts in the proportional abundance of several taxa. Mediterranean diet-fed NHPs with lower body fat had a greater proportion of *Lactobacillus animalis* than their higher body fat counterparts. Higher body fat Western diet-fed NHPs had more *Ruminococcus champaneliensis* and less *Bacteroides uniformis* than their low body fat counterparts. Western diet-fed NHPs had significantly higher levels of *Prevotella copri* than Mediterranean diet NHPs. Western diet-fed subjects were stratified by *P. copri* abundance (*P. copri*^HIGH^ versus *P. copri*^LOW^), which was not associated with adiposity. Overall, Western diet-fed animals in the *P. copri*^HIGH^ group showed greater proportional abundance of *B. ovatus*, *B. faecis*, *P. stercorea*, *P. brevis*, and *Faecalibacterium prausnitzii* than those in the Western *P. copri*^LOW^ group. Western diet *P. copri*^LOW^ subjects had a greater proportion of *Eubacterium siraeum*. *E. siraeum* negatively correlated with *P. copri* proportional abundance regardless of dietary consumption. In the Western diet group, Shannon diversity was significantly higher in *P. copri*^LOW^ when compared to *P. copri*^HIGH^ subjects. Furthermore, gut *E. siraeum* abundance positively correlated with HDL plasma cholesterol indicating that those in the *P. copri*^LOW^ population may represent a more metabolically healthy population. Untargeted metabolomics on urine and plasma from Western diet-fed *P. copri*^HIGH^ and *P. copri*^LOW^ subjects suggest early kidney dysfunction in Western diet-fed *P. copri*^HIGH^ subjects.

**Conclusions:**

In summary, the data indicate diet to be the major influencer of gut bacterial diversity. However, diet and adiposity must be considered together when analyzing changes in abundance of specific bacterial taxa. Interestingly, *P. copri* appears to mediate metabolic dysfunction in Western diet-fed NHPs.

**Video abstract**

**Supplementary Information:**

The online version contains supplementary material available at 10.1186/s40168-021-01069-y.

## Background

Gut microbiome dysbiosis is associated with many adverse health outcomes including multiple sclerosis, cancer, diabetes (types 1 and 2), asthma, allergies, inflammatory bowel disease, obesity, and autism [1–6]. Composition of the gut microbiome is influenced by several factors including maternal delivery method, ethnicity, geography, and lifestyle [7]. Of the multiple factors associated with the term “lifestyle,” diet in particular has a major influence on the microbial composition within the gut. Bacterial abundance is modulated by dietary macronutrient consumption, including proteins, carbohydrates, and fats [6, 8, 9]. Poor diet and obesity often are linked, making it difficult to determine which variable is the major driver of the gut microbiome composition.

The composition of the human gut microbiome is dominated by the Bacteroidetes and Firmicutes bacterial phyla [10]. Within the Bacteroidetes phylum, enterotypes are distinguished by abundance of *Bacteroides* or *Prevotella*. While both *Prevotella* and *Bacteroides* are saccharolytic bacteria, these genera of bacteria tend to inhibit each other, thereby giving way to two separate enterotypes [11]. The prevalence of either strongly associates with long-term diets; *Bacteroides* predominate with protein and animal fat consumption and *Prevotella* are observed with carbohydrate (fiber) consumption [12]. Enteric *Prevotella* are more common in non-Westernized populations consuming a plant-rich diet or in Western populations with high adherence to a Mediterranean or vegetarian diet [13]. Food and nutrient consumption patterns, such as omnivore versus plant-based diet, are associated with shifts in the human gut microbiome. Subjects consuming an omnivore diet displayed increased *Ruminococcus* and *Streptococcus* abundance, while subjects on a vegetable-based diet showed increased abundance of *Roseburia*, *Lachnospira*, and *Prevotella* [14]. High-fat diet and obesity are linked to modulation of these phyla in humans and mice; both factors are associated with increased Firmicutes and decreased Bacteroidetes phyla in the gut metagenome [6, 8, 9, 15–17].

The microbiome of non-human primates (NHP) harbors Bacteroidetes, Firmicutes, and Proteobacteria, similar to the human microbiome [18, 19]. Prominent differences (see [19]) exist between the macaques and human microbiome such as elevated *Helicobacter* and *Spirochaetes* in NHPs compared to humans, albeit these NHPs were consuming standard adult monkey chow that would not compare to humanized dietary patterns and may account for some of the noted differences [19]. A study investigating the differences in the gut microbiome between captive and wild NHPs (8 different species) indicate that captivity “humanizes” the primate microbiome [20]. Taken together, these data suggest that application of a humanized dietary pattern in captive NHPs may accurately represent the human microbiome. As an additional benefit, the establishment of a NHP model allows for a closely controlled study without adherence or self-reporting issues, and avoiding medication, all of which often are confounding factors in human studies.

This study examines the impact of a translationally relevant humanized Western and Mediterranean dietary patterns on the gut microbiota, and association with obesity, in female middle-aged cynomolgous macaques. Application of these diets has been shown to significantly influence the gut populations of cynomolgous macaques [18]. Mediterranean diet consumption increased the genera *Lactobacillus*, *Clostridium*, *Faecalibacterium*, *and Oscillospira* while decreasing *Ruminococcus* and *Coprococcus* representation [18]. However, the aforementioned report relied on 16S rRNA sequencing. In the study reported here, we instead utilize metagenomic sequencing, the methodology used in Human Microbiome Project [21]. 16S sequencing is useful in studies where many samples are available, but has poor resolution compared to metagenomics [22]. Shotgun metagenomic sequencing offers enhanced functional and taxonomic resolution to allow identification of specific bacterial species and strains [21–23]. This has allowed us to report bacterial species as well as virulence factor clusters modulated by dietary exposure in cynomolgous macaques, improving understanding of the similarities and differences between NHPs and humans with the respect to influence of diet on the gut microbiome. In addition, we assessed complete metabolic profiling data for each of the animals to correlate gut microbiota populations with body weight (BW), body adiposity, plasma cholesterol levels, insulin tolerance testing, and metabolite signatures in plasma or urine. This enabled the identification of specific microbes that may potentially influence metabolic syndrome development.

## Methods

### NHP subjects

Adult female *Macaca fascicularis* were obtained (SNBL USA, Ltd., Alice, Texas) and housed in groups of 4 animals per pen with daylight exposure on a 12/12 light/dark cycle. Animals were aged at study initiation by dentition to be approximately 8.8 years old. The subjects were habituated to their social groups and were metabolically characterized for group randomization during a 7-month baseline phase while consuming standard monkey chow (monkey diet 5037/5038; LabDiet, St. Louis, Missouri). Animal groups were randomized so that no differences in age, body weight (BW), body mass index (BMI), or plasma triglyceride concentrations were observed. No gastrointestinal differences or other health concerns were observed at baseline before animals were randomized to dietary pattern. Animals were then assigned to a dietary pattern (Western *n*=21 or Mediterranean *n*=17) for 31 months. All animal manipulations were performed according to the guidelines of state and federal laws, the US Department of Health and Human Services, and the Animal Care and Use Committee of Wake Forest University School of Medicine.

### Diet formulation

Experimental diets were formulated to be isocaloric with respect to protein, fat, carbohydrates, and cholesterol content. Experimental diets translationally represent humanized Western and Mediterranean dietary patterns. For further details on diet formulation and ingredients, see Supplemental Table 1 and references [24, 25].

### Metabolic characterization

Metabolic parameters were measured in each subject and previously reported [24, 25]. Briefly, BW (kg) and body length (BL, m) were measured throughout the study. BMI was calculated as BW/(BL)^2^. BW and BMI reported in Table 1 were measured at the endpoint of the dietary intervention study (31 months) in all subjects and subjects were categorized into tertiles by BMI. For this particular analysis, only animals classified in the lowest (*N*=14) or highest (*N*=15) tertiles underwent microbiome analysis and comprise the sample included in this report. Body composition was measured by computed tomography in anesthetized subjects during various intervals throughout the study. Body fat composition reported in Table 1 was measured at month 27 of the treatment phase. The fat compartment was defined as tissue with attenuation between −190 and −30 Hounsfield units, and total fat was then determined across the whole body. Intravenous glucose tolerance tests with insulin responses were performed during month 26 of the treatment phase as previously described [18]. In brief, subjects were fasted for 18 h, sedated with ketamine HCl (15 mg/kg), and dosed with 500 mg/kg dextrose. Blood samples were taken at 0, 5, 10, 20, 30, 40, and 60 min. Insulin area under the curve (AUC) was calculated using insulin responses between 10 and 40 min. Insulin was determined using a commercial enzyme-linked immunosorbent assay (ELISA; Mercodia, Uppsala, Sweden).

**Table 1:** Metabolic parameters by body weight group. Values represent mean ± standard deviation

	Mediterranean-lean (*n*=7)	Mediterranean-heavy (*n*=7)	Western-lean (*n*=7)	Western-heavy (*n*=8)
BW (kg)	**2.5±0.2**	**4.1±1.0**	**2.7±0.2**	**4.9±1.2**
BMI (kg/m^2^)	**36.7±4.2**	**48.2±6.9**	**39.7±4.5**	**60.2±10.4**
Body fat composition (%)	**8.4±2.0**	**23.4±9.0**	**11.7±4.3**	**39.1±10.5**
Insulin AUC	**1721±335**	**6456±5703**	**3431±4043**	**10526±8427**
TPC (mg/dL)	**133.4±29.4**	**163.7±48.0**	**159.5±33.1**	**149.9±28.5**
HDL-C (mg/dL)	**48.6±8.6**	**66.7±29.8**	**78.4±40.6**	**72.1±17.2**
TPC/HDL-C ratio	**2.78±0.50**	**2.62±0.46**	**2.38±0.95**	**2.13±0.16**
Cortisol (µg/dL)	**36.5±4.2**	**29.3±5.6**	**33.7±8.8**	**41.1±5.7**

Total plasma cholesterol (TPC) and high-density lipoprotein cholesterol (HDL-C) were measured at 24 months from plasma collected after an 18 h fast. TPC and HDL-C levels were determined by the Wake Forest Comparative Medicine Clinical Chemistry and Endocrinology Laboratory using reagents (ACE cholesterol and ACE HDL-C standards) and instrumentation (ACE ALERA auto analyzer) from Alfa Wasserman Diagnostic Technologies (West Caldwell, NJ). TPC and HDL-C were standardized to calibrated controls from the Centers for Disease Control and Prevention/National Institutes of Health Lipid Standardization Program [26]. Blood samples for cortisol assay were drawn in the morning within 9 min of staff entering NHP housing, and serum was assayed with RIA kits from DiaSource (IBL America, Minneapolis, MN).

### Fecal and urine sample collection

Urine and fecal samples were collected after 26 months of dietary treatments. Animals were moved to metabolic cages with wire bottoms, and sample cups were checked every 15 min for feces. Fecal samples were immediately placed in sterile tubes under aseptic conditions and stored at −80 °C until further processing. As soon as adequate urine samples were available, they were collected, and stored at −80 °C until analysis.

### Metagenomic sequencing

DNA was isolated from 100 mg of frozen feces using the Qiagen DNeasy PowerSoil Pro kit (Valencia, CA), and metagenomic sequencing was performed by CosmosID Inc. (Rockville, MD). In brief, DNA libraries were prepared using the Illumina Nextera XT library preparation kit (San Diego, CA), with a modified protocol [27, 28]. Library quantity was assessed with a Qubit Fluorometer (Thermo Fisher Scientific, Wilmington, DE). Libraries were then sequenced on an Illumina HiSeq platform to generate 150-bp paired-ends reads.

### Metagenomic bioinformatic analysis

Unassembled sequencing reads (≥12M read depth; see Supplemental Figure S1 for individual sample read statistics) were analyzed using the CosmosID bioinformatics platform described elsewhere [29–32] for multi-kingdom microbiome analysis, profiling of antibiotic resistance and virulence genes, and quantification of microbial relative abundance. Briefly, the system utilizes curated genome databases and a high-performance data-mining algorithm that rapidly disambiguates hundreds of millions of metagenomic sequence reads into the discrete microorganisms engendering the particular sequences. CosmosID bioinformatics utilizes high performance data mining algorithms and highly curated dynamic comparator databases (GenBook®). GenBook® comprises 150,000+ bacteria, viruses, fungi and protist genomes, and gene sequences. The GenBook database is organized as phylogenetic tree and comprised of libraries of hundreds of millions of marker sequences, representing both coding and noncoding sequences shared or uniquely identified across different taxa and/or distinct nodes of phylogenetic trees. Comparative metagenomic analyses (principal component analysis, double hierarchical clustering, centroid classification, and other statistical analyses) were done to determine temporal changes, geographical diversity, and shifts in diversity correlated with treatment and to differentiate datasets of the different cohorts. Monkey group housing (pen effect) had no significant effect on microbiota populations (Supplemental Figure S2).

### Metabolomics

Untargeted metabolomics from urine and plasma samples collected at 26 months were performed by Metabolon (Raleigh, NC) as previously described [24]. In brief, all experiments used a Waters ACQUITY ultra-performance liquid chromatography (UPLC) and a Thermo Scientific Q-Exactive high resolution/accurate mass spectrometer interfaced with a heated electrospray ionization (HESI-II) source and Orbitrap mass analyzer operated at 35,000 mass resolution. The scan range varied slightly between methods but covered 70-1000 m/z. Raw data were extracted, peak- identified, and processed for quality control using Metabolon’s hardware and software. Compounds were identified by comparison to library entries of purified standards or recurrent unknown entities. Peaks were quantified using area-under-the-curve. The informatics consisted of the Laboratory Information Management System (LIMS), the data extraction and peak-identification software, data processing tools for quality control and compound identification, and a collection of information interpretation and visualization tools for use by data analysts. The hardware and software foundations for these components were the LAN backbone, and a database server running Oracle 10.2.1.1 Enterprise Edition. Values were then log transformed, and missing values, if any, were imputed with the minimum observed value for each compound.

### Statistical analysis

Metabolic parameters were summarized using means and standard deviations. Microbiome diversity and bacterial populations were compared by diet group using two group *t* tests allowing for unequal variance using the Satterthwaite method for degrees of freedom. Principal coordinates analysis (PCoA) of bacterial beta diversity based on the Bray-Curtis dissimilarity matrix using relative abundance was used to distinguish groups. Diversity analyses were performed using species taxonomy level. Comparisons by diet and body weight group were assessed by two-way ANOVA of diet, group, and their interaction, estimating different variance components by diet and group; we used linear contrasts to make pairwise comparisons within diet group if the interaction *p* value was <0.10. Within the Western diet group, animals were split into *P. copri*^LOW^ and *P. copri*^HIGH^ groups based on whether they were below the median, and Welch’s two-sample *t* test was used to compare microbiota and metabolites shifts between Western diet *P. copri*^HIGH^ and Western diet *P. copri*^LOW^ experimental groups. Correlations between gut microbial populations and metabolic parameters were summarized by Pearson correlation and expressed as *r*. A *p* value of *p*<0.05 was considered statistically significant in all analyses with the exception of the evaluation of diet × group interaction in the two-way ANOVA which used alpha=0.10.

## Results

**Fig. 1:**
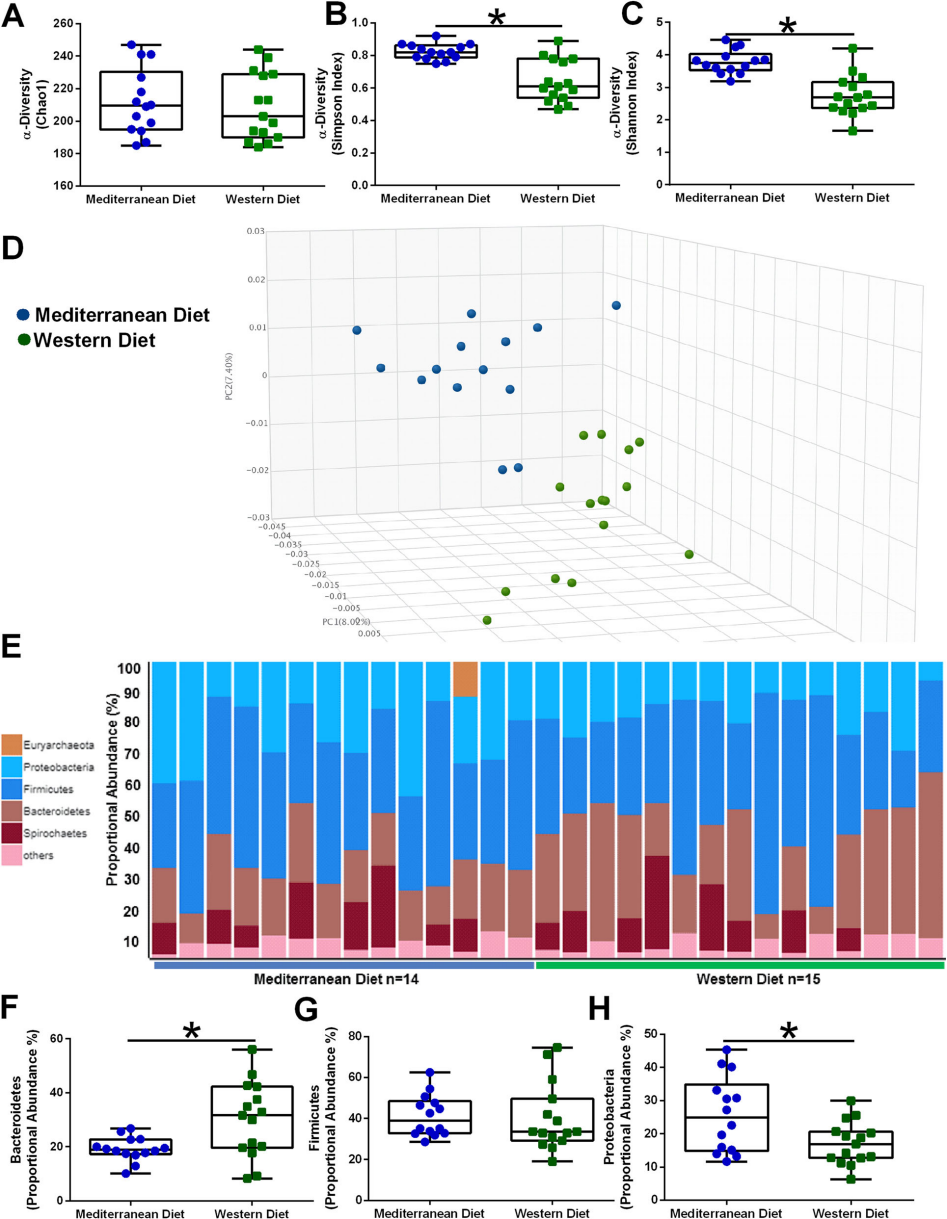
Diet is a driver of gut microbiome diversity. Alpha diversity was estimated with the Chao1 index (**a**), Simpson index (**b**), and Shannon index (**c**) on raw OTU abundance in Mediterranean diet and Western diet-fed subjects. **d** Principal coordinates analysis (PCoA) of bacterial beta diversity based on the Bray-Curtis dissimilarity of gut microbial populations by relative abundance indicates that subjects separate by dietary pattern consumption. Mediterranean diet-fed subjects are shown in blue, while Western diet-consuming animals are shown in green. **e** Relative abundance of bacterial phyla in different fecal samples is visualized by bar plots. Each bar represents a subject and each colored box a bacterial phylum. The height of a color box represents the relative abundance of that organism within the sample. “Other” represents lower abundance taxa. **f** Western diet-fed NHPs displayed higher Bacteroidetes. **g** Diet had no significant effects on shifting Firmicute abundance. **h** Mediterranean diet-fed subjects showed higher proportional abundance of Proteobacteria. *n*=14-15; *p<0.05. Error bars in box plots show the min to max distribution

Western and Mediterranean dietary pattern drives gut microbiome populations. While dietary pattern consumption had no significant effects on bacterial richness (Choa1; Fig. 1a), subjects fed a Mediterranean diet displayed higher microbial diversity as indicated by Simpson index (Fig. 1b) and Shannon diversity score (Fig. 1c). PCoA of gut microbial populations by relative abundance indicates that subjects separate by dietary pattern consumption (Fig. 1d). The bulk of the microbial biomass at the phyla level are derived from Bacteroidetes (20-40%), Firmicutes (40%), and Proteobacteria (15-30%). Proportional abundance of each phylum within each animal is shown as bar graph (Fig. 1e). Consumption of a Western diet significantly elevated Bacteroidetes abundance (Fig. 1f), but had no effect on Firmicutes populations (Fig. 1g). Mediterranean diet-fed subjects displayed increased Proteobacteria abundance when compared with Western diet-fed animals (Fig. 1h).

**Fig. 2:**
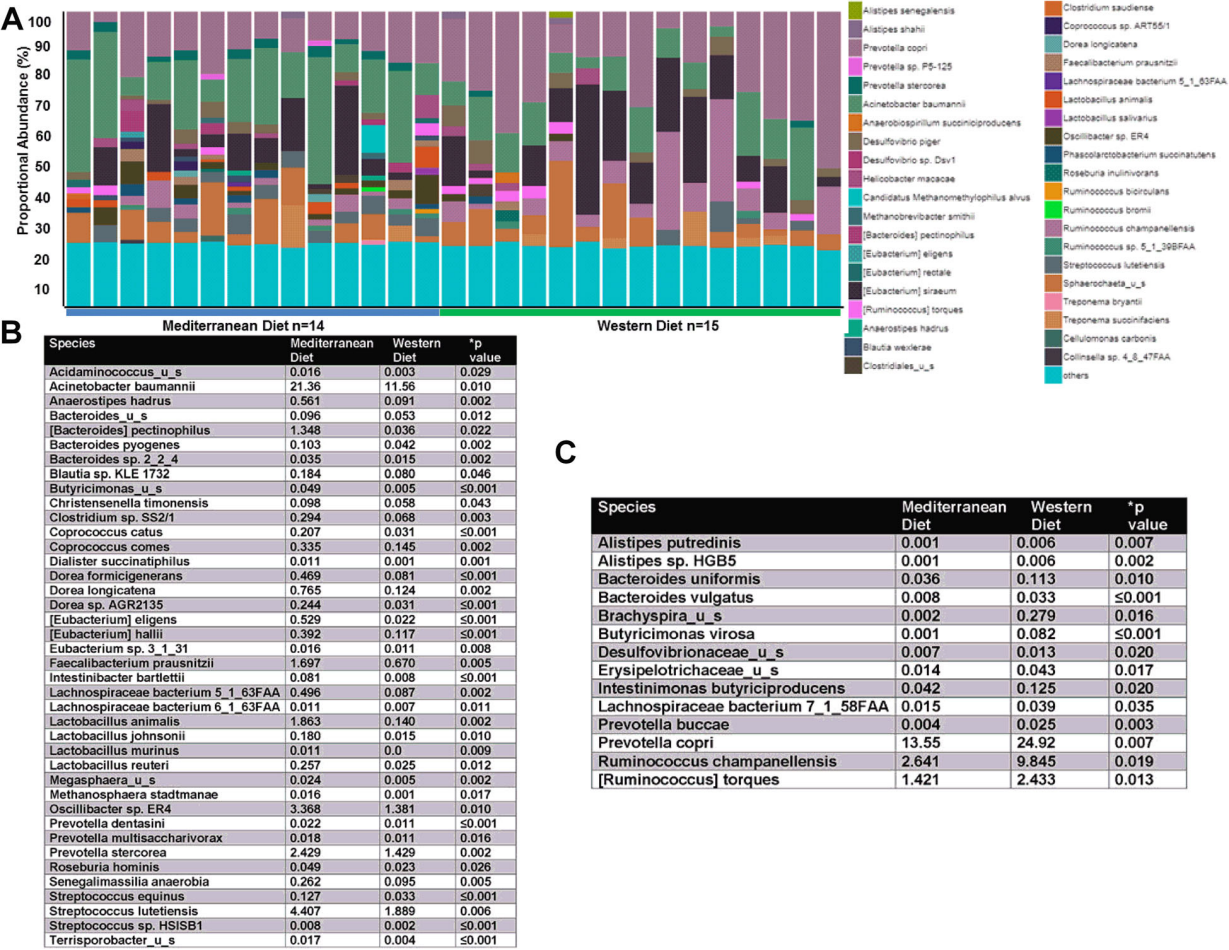
Consumption of Mediterranean diet leads to significant variation in distinct gut microbiota species. **a** Relative abundance of bacterial species in different fecal samples is visualized by bar plots. Each bar represents a subject and each colored box a bacterial taxon. The height of a color box represents the relative abundance of that organism within the sample. “Other” represents lower abundance taxa. Mediterranean diet-fed subjects are shown in blue; Western diet-fed subjects are shown in green. **b** Species elevated in Mediterranean diet-consuming animals when compared with Western diet-fed subjects (mean values and *p* values) are shown in the table. **c** Species elevated in Western diet-fed animals when compared with Mediterranean diet-consuming subjects (mean values and *p* values) are shown in the table

At the species level, 507 different species of bacteria were detected in the fecal samples obtained from diet-treated subjects. The proportional abundance of the more abundant species is shown in Fig. 2a in which each bar represents a fecal sample from an individual subject. Diet significantly shifted the proportional abundance of 54 different species. See Fig. 2b-c for all 54 species. Of specific interest, Mediterranean diet-fed subjects displayed increased proportional abundance of *Bacteroides* (*B.*) *pectinophilus*, *Clostridium* sp. *SS2/1*, *Coprococcus catus*, *Coprococcus comes*, *Dorea formicigenerans*, *Dorea longicatena*, *Eubacterium* (*E.*) *eligens*, *E. hallii*, *Faecalibacterium prausnitzii*, *Lachnospiraceae bacterium 5_1_63FAA*, *Lachnospiraceae bacterium 6_1_63FAA*, *Lactobacillus* (*L.*) *animalis*, *L. johnsonii*, *L. murinus*, *L. reuteri*, *Oscillibacter* sp. *ER4*, *Prevotella* (*P.*) *dentasini*, *P. stercorea*, *Roseburia hominis*, and *Streptococcus lutetiensis* (Fig. 2b). Western diet-fed subjects displayed elevated proportional abundance of *Alistipes putredinis*, *B. uniformis*, *B. vulgatus*, *Butyricimonas virosa*, *Lachnospiraceae bacterium 7_1_58FAA*, *P. buccae*, *P. copri*, *Ruminococcus champanellensis*, and *Ruminococcus torques* (Fig. 2c). While 44 of these diet-associated species have less than a 1% proportional abundance within the samples, 10 species display up to 25% abundance within the sample.

**Fig. 3:**
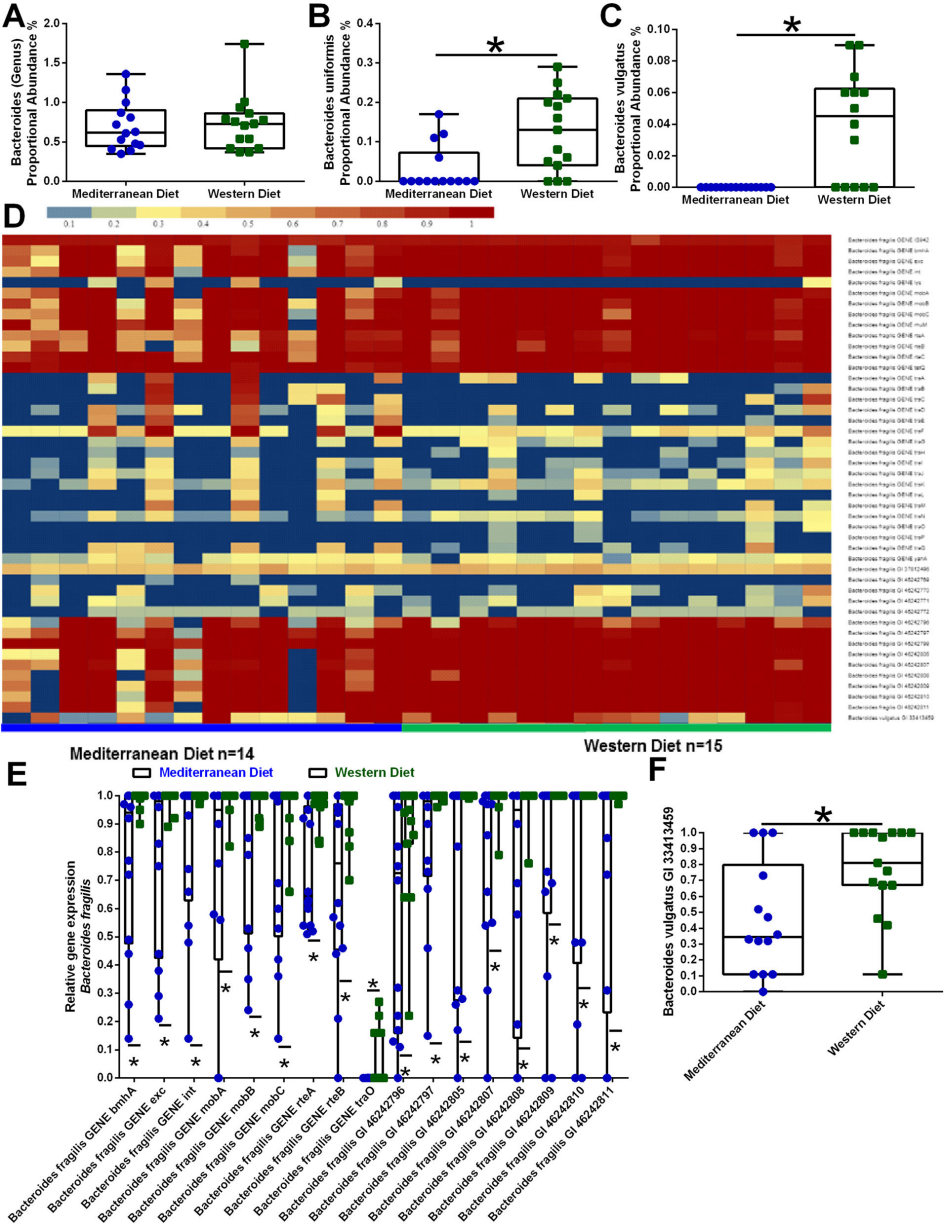
Virulence factor clustering analysis highlights Bacteroides populations varied by dietary consumption. **a** At the genus level, Bacteroides did not differ by diet (*p*=0.79). *Bacteroides uniformis* (**b**) and *Bacteroides vulgatus* (**c**) populations were elevated in Western diet fed NHPs. Furthermore, virulence factor measurements from the shotgun metagenomics sequencing highlighted increased *Bacteroides fragilis* (**d** and **e**) and *Bacteroides vulgaris* (**f**) in Western diet-consuming NHPs. *n*=14-15. *p<0.05 using two group *t* test for unequal variance with Satterthwaite adjustment. Error bars on box plots show the min to max distribution

While not a major component of the NHP gut microbiota (≤1%), *Bacteroides* genus did not differ by diet (Fig. 3a), distinct *Bacteroides* species differed by dietary pattern consumption. *B. uniformis* (Fig. 3b, *p*=0.005) and *B. vulgatus* (Fig. 3c, *p*<0.001) were higher in animals fed the Western diet than the Mediterranean diet. Furthermore, virulence factor gene measurements from the shotgun metagenomics sequencing highlighted elevated *B. fragilis* (Fig. 3d and e) and *B. vulgaris* (Fig. 3d and f) in Western diet consuming NHPs (all *p* values <0.05).

Shotgun metagenomics sequencing allows for identification of viruses, fungi, protozoa, and phages in addition to the more commonly investigated bacteria populations. We show that dietary patterns also modulate gut viral and protist communities with no effect on fungal or phage populations (Supplemental Figure S3). There were approximately 32 different viral species identified in the NHP fecal samples, of which five species were associated by diet. Western diet-fed NHP displayed elevated fecal baboon endogenous virus strain M7 and RD114 retrovirus. Mediterranean diet-fed NHP displayed elevated fecal human mastadenovirus G, Sfi11 virus u_s, and Streptococcus virus 7201 (Supplemental Figure S3A). In the protozoa community, approximately 12 species were identified in the NHP fecal samples. Western diet-fed NHP displayed elevated fecal *Endolimax nana* abundance and reduced *Neobalantidium coli* abundance when compared with fecal samples obtained from Mediterranean diet-fed NHPs (Supplemental Figure S3B).

**Fig. 4:**
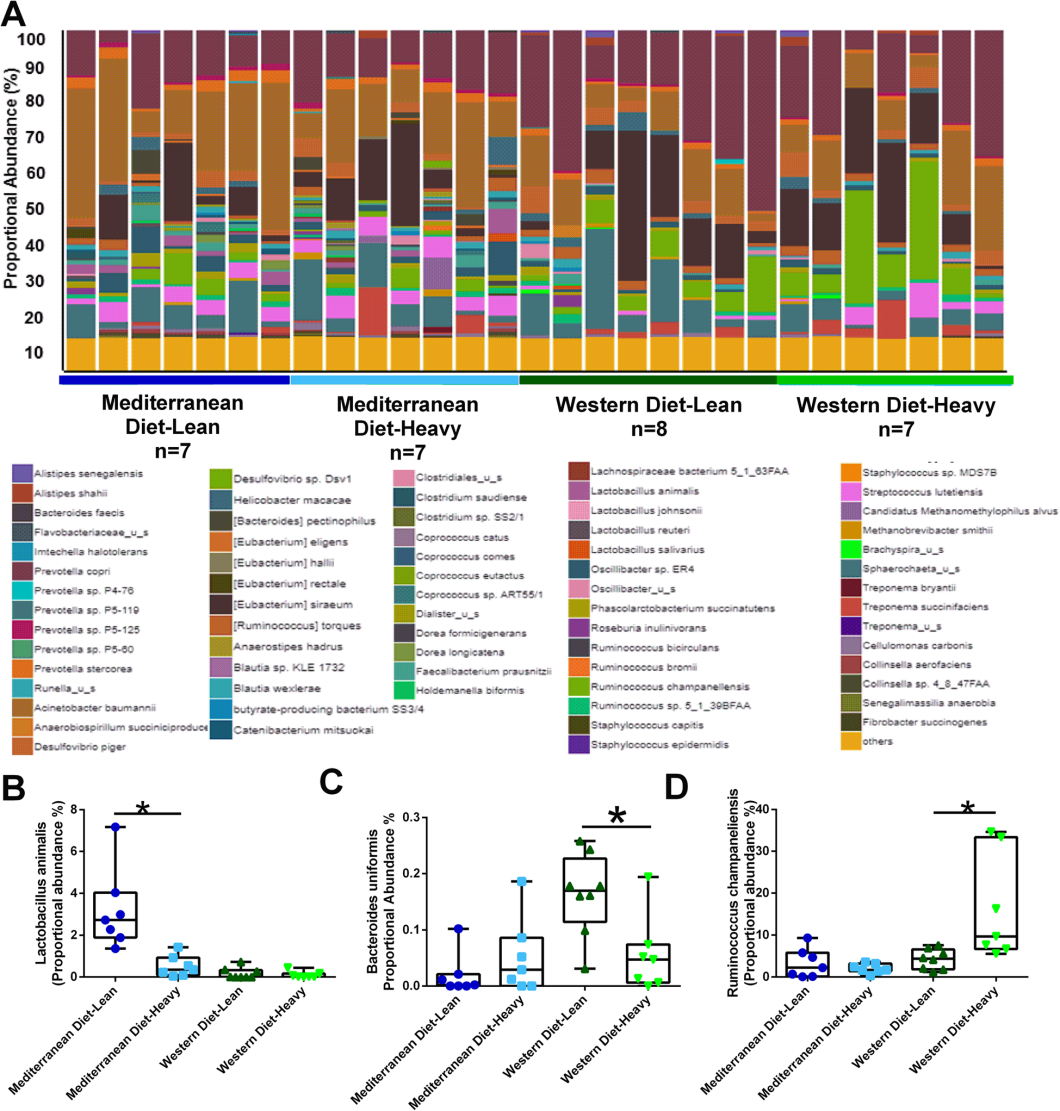
Body adiposity shifts gut microbiota patterns within dietary patterns. **a** Relative abundance of bacterial species in different fecal samples is visualized by bar plots. Each bar represents a subject and each colored box a bacterial taxon. The height of a color box represents the relative abundance of that organism within the sample. “Other” represents lower abundance taxa. Lean Mediterranean diet-fed subjects are shown in dark blue; heavy Mediterranean diet-fed subjects are shown in light blue; lean Western diet-fed subjects are shown in dark green; and heavy Western diet-fed animals are shown in light green. **b** Lean Mediterranean diet-fed animals display higher *Lactobacillus animalis* abundance when compared to heavy Mediterranean diet-fed subjects, lean Western diet-fed subjects, and heavy Western diet-fed subjects. Lean Western diet-fed monkeys displayed increased gut abundance of *Bacteroides uniformis* (**c**) when compared to heavy animals fed the same diet. Heavy Western diet-fed subjects displayed increased *Ruminococcus champaneliensis* (**d**) when compared to the relative abundance of these species within lean Western diet-fed NHPs. *n*=7-8. *p from pairwise comparison in two-way ANOVA diet × group interaction used alpha=0.10. Error bars show the min to max distribution

Adiposity within dietary pattern consumption cohort shifts differential microbiota abundance. Some of the effect of dietary pattern on gut microbiota species differed by body adiposity. The gut microbiota species within each subject is shown in a bar graph of proportional abundance with each bar representing an individual subject and separate colors for distinct bacterial species (Fig. 4a). Specifically, there was a significant interaction between diet and adiposity. Microbe abundance within dietary patterns differed by adiposity for *L. animalis* (Fig. 4b, *p* interaction=0.005), *B. uniformis* (Fig. 4c, *p*=0.064), and *Ruminococcus champaneliensis* (Fig. 4d, *p*=0.034). Within Mediterranean diet-fed subjects, monkeys with increased body adiposity displayed decreased *L. animalis* (Fig. 4b) when compared to their leaner Mediterranean diet-fed counterparts. Adiposity did not regulate *L. animalis* in Western diet-fed animals. Lean Western diet-fed monkeys had increased *B. uniformis* (Fig. 4c) when compared with the proportional abundance in heavier Western diet-fed animals, but Mediterranean diet-fed animals did not have different levels of abundance by adiposity. Heavier Western diet consuming subjects displayed elevated *Ruminococcus champaneliensis* (Fig. 4d) compared to lean Western diet-fed animals, but Mediterranean diet-fed animals did not have different levels of abundance by adiposity.

**Fig. 5:**
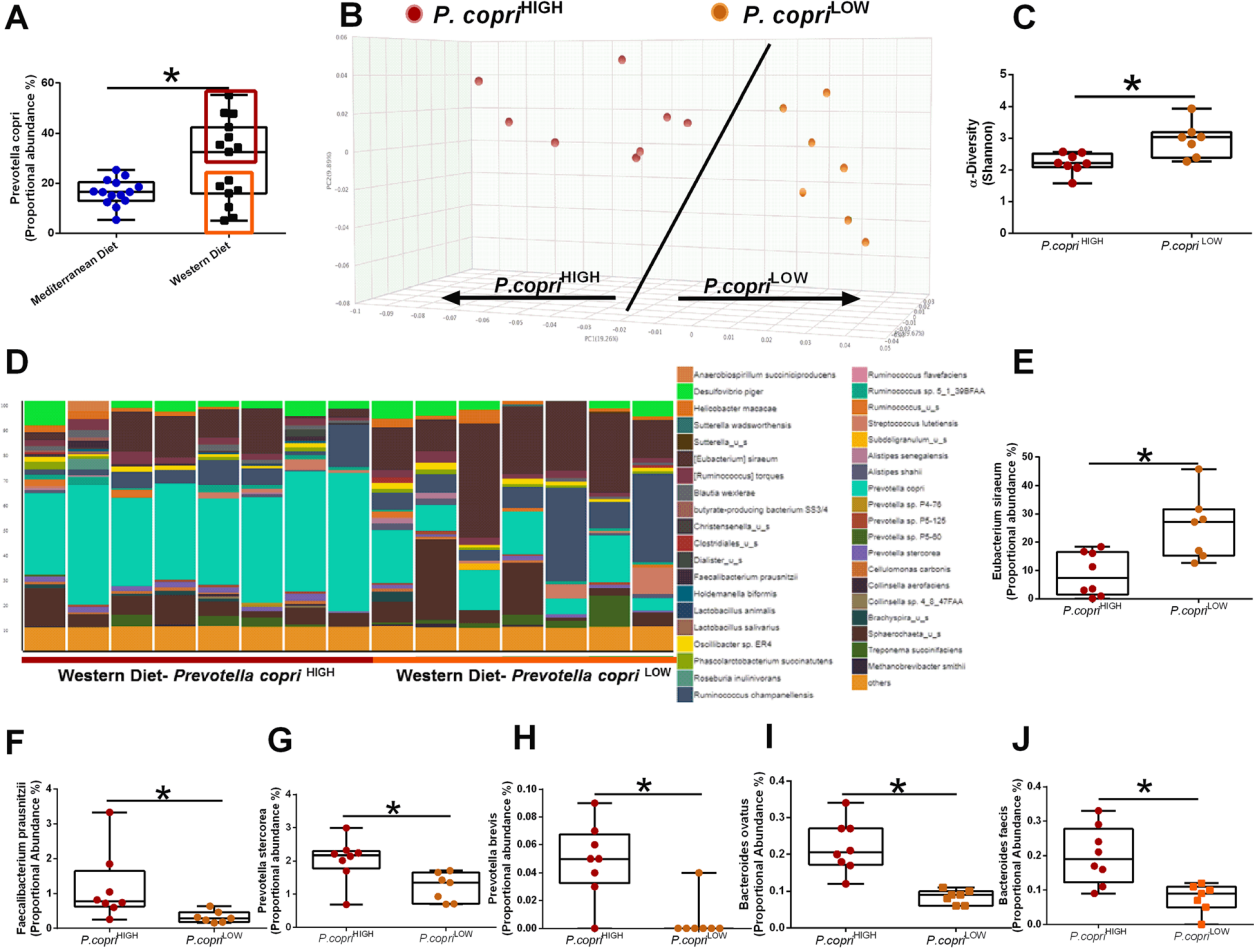
Western diet-fed subjects cluster into two different enterotypes based upon *Prevotella copri* abundance. **a** Proportional abundance of *Prevotella copri* in Mediterranean and Western diet-fed NHPs. *n* = 14–15, *p < 0.05 from Welch’s two group *t* test. Error bars show the min to max distribution. **b** Principal coordinates analysis (PCoA) of bacterial beta diversity based on the Bray-Curtis dissimilarity using relative abundance. *P. copri*^HIGH^ samples (*n*=8) are shown in red circles and *P. copri*^LOW^ samples are shown in orange circles. **c** Alpha diversity was estimated with the Shannon index on raw OTU abundance based upon *P. copri* abundance. Shannon diversity is significantly higher in *P. copri*^LOW^ animals. **d** Relative abundance of bacterial species in different fecal samples is visualized by bar plots. Each bar represents a subject and each colored box a bacterial taxon. The height of a color box represents the relative abundance of that organism within the sample. “Other” represents lower abundance taxa. **e** Proportional abundance of *Eubacterium siraeum* is elevated in *P. copri*^LOW^ subjects. *P copri*^HIGH^ samples are characterized by elevated *Faecalibacterium prausnitzii* (**f**), *Prevotella stercorea* (**g**), *Prevotella brevis* (**h**), *Bacteroides ovatus* (**i**), and *Bacteroides faecis* (**j**). *n*=6-8; *p<0.05 from Welch’s two group *t* test

*P. copri* enterotypes categorize two distinct populations within Western diet consuming subjects. NHPs consuming a Western diet display elevated abundance of *P. copri* when compared with Mediterranean diet-fed animals (Fig. 5a). Within Western diet-fed animals, subjects could be stratified into two distinct enterotypes: *P. copri*^HIGH^ and *P. copri*^LOW^. As shown in the PCoA (Fig. 5b), the two enterotypes are distinct from each other as indicated by increased distance between clusters. Shannon diversity was reduced in the Western diet-fed *P. copri*^HIGH^ group (Fig. 5c). Animals in the *P. copri*^LOW^ group displayed elevated *E. siraeum* (Fig. 5d and e). Animals in the *P. copri*^HIGH^ displayed increased *Faecalibacterium prausnitzii* (Fig. 5f), *P. stercorea* (Fig. 5g), *P. brevis* (Fig. 5h), *B. ovatus* (Fig. 5i), and *B. faecis* (Fig. 5j).

**Fig. 6:**
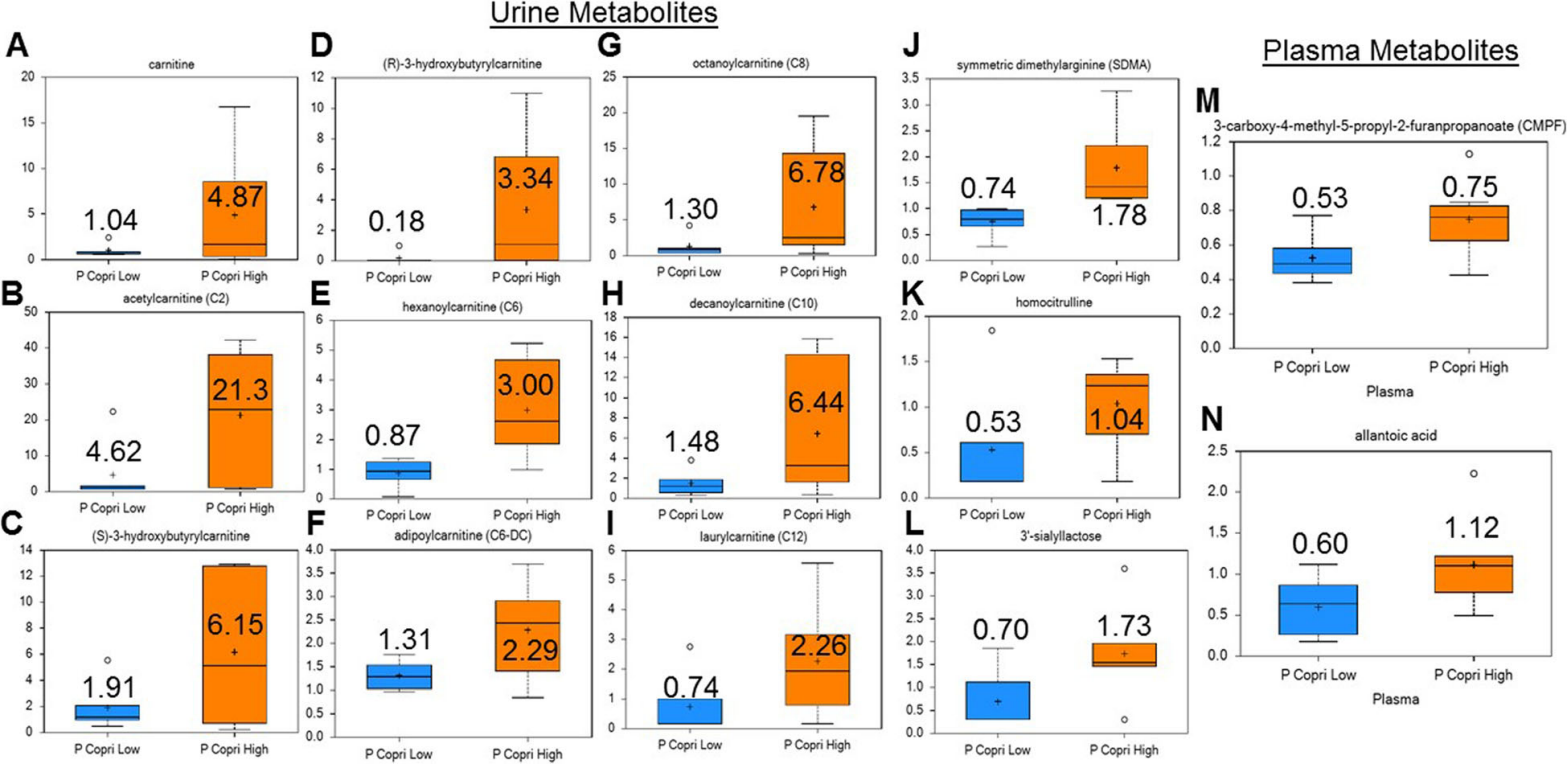
Western diet-fed subjects display differential urine and plasma metabolites regulation based upon gut *Prevotella copri* abundance. Urinary biomarkers elevated in Western diet-fed *P. copri*^HIGH^ subjects include carnitine-based metabolites (carnitine, acetylcarnitine, (S)-3-hydroxybutyrylcarnitine, (R)-3-hydroxybutyrylcarnitine, adipoylcarnitine (C6-DC), hexanoylcarnitine (C6), octanoylcarnitine (C8), decanoylcarnitine (C10), and laurylcarnitine (C12)), uremic toxin symmetric dimethylarginine (SDMA), homocitrulline, and 3′-sialyllactose. Plasma levels of 3-carboxy-4-methyl-5-propyl-2-furanpropanoate (CMPF) and allantoic acid were elevated between Western diet-fed *P. copri*^HIGH^ and *P. copri*^LOW^ subjects. *n* = 6-8, *p* < 0.05 from Welch’s two group *t* test. Error bars show the min to max distribution; the mean value is displayed within/next to whisker plot

Untargeted metabolomics was performed on urine and plasma samples from Western diet-fed *P. copri*^HIGH^ and *P. copri*^LOW^ subjects. Urinary biomarkers elevated in Western diet-fed *P. copri*^HIGH^ subjects (Fig. 6) include carnitine-based metabolites (carnitine, acetylcarnitine, (S)-3-hydroxybutyrylcarnitine, (R)-3-hydroxybutyrylcarnitine, adipoylcarnitine (C6-DC), hexanoylcarnitine (C6), octanoylcarnitine (C8), decanoylcarnitine (C10), and laurylcarnitine (C12)), uremic toxin symmetric dimethylarginine (SDMA), homocitrulline, and 3′-sialyllactose. While urinary levels of 3-carboxy-4-methyl-5-propyl-2-furanpropanoate (CMPF) and allantoic acid were not different between Western diet-fed *P. copri*^HIGH^ and *P. copri*^LOW^ subjects, plasma levels of CMPF and allantoic acid were significantly higher in the *P. copri*^HIGH^ group. *P. copri*^LOW^ subjects displayed elevated levels of plasma gamma glutamyl amino acids and phosphodtidylethanolamine (PE) metabolites (Supplemental Figure S4), suggesting potential lipid and amino acid metabolism regulation.

**Fig. 7:**
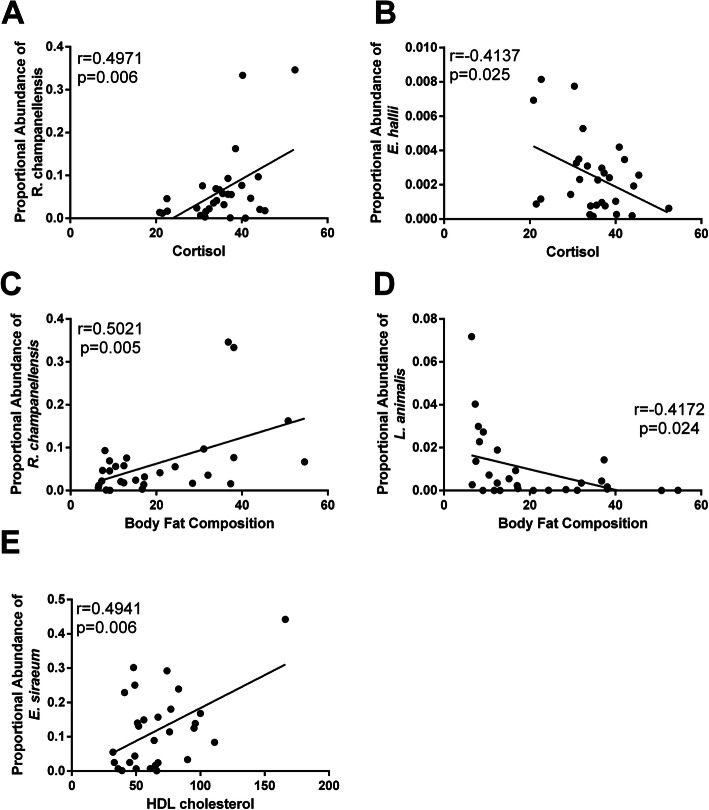
Certain microbiota species correlate with metabolic parameters. **a** Serum cortisol levels positively correlates with gut *Ruminococcus champanellensis* abundance. **b** Serum cortisol levels negatively correlates with gut *Eubacterium hallii* abundance. **c** Gut *Ruminococcus champanellensis* abundance correlates with increasing % body fat composition. **d** Proportional abundance of *Lactobacillus animalis* negatively correlates with % body fat composition. **e** Gut *Eubacterium siraeum* correlates with plasma HDL cholesterol levels. *n*=29; Pearson correlation coefficient (*r*)

Gut microbiota populations correlate with metabolic parameters. Regardless of dietary pattern consumption, certain gut microbiota populations correlated with plasma cortisol levels; gut *Ruminococcus champanellensis* positively correlated with plasma cortisol (Fig. 7a) and with percent body fat composition (Fig. 7c), while *E. hallii* abundance negatively correlated with plasma cortisol (Fig. 7b). Furthermore, we observed *L. animalis* abundance negatively correlated with percent body fat composition (Fig. 7d) and gut *E. siraeum* positively correlated with plasma HDL levels (Fig. 7e).

## Discussion

Our data indicates that diet composition impacts gut microbiota structure and that a Mediterranean diet pattern is associated with increased gut bacterial diversity in female cynomolgous macaques. In accordance with human diet studies in which subjects with high adherence to a Mediterranean diet display elevated Proteobacteria populations [33], Mediterranean diet-fed NHPs also show increased Proteobacteria phyla proportional abundance in response to this diet. While NHPs have similar gut microbiota populations in comparison to humans [34] and respond to translationally relevant diets concordantly in regards to alpha diversity, the response to Western diet at the phyla level appears to differ. In humans, consumption of high fat animal-based Westernized diet leads to decreased Bacteroidetes: Firmicutes ratio, mainly due to enrichment of Firmicutes. We confirmed previous results from 16S sequencing data demonstrating that Western diet-fed NHPs displayed increased proportional abundance of Bacteroidetes phylum with no significant shifts in Firmicutes populations [18]. Consumption of a Western-like diet in the African green monkey led to a similar outcome, although important to note that this study relied on 16S sequencing and was unable to identify species level classifications [35]. The elevated Bacteroidetes abundance observed in the NHPs is driven by an enrichment in *P. copri*. Gut *P. copri* displays an interesting case of duality in human health and disease. Non-westernized lifestyle is associated with increased gut *P. copri* abundance [36]. Literature strongly supports the association of gut *P. copri* abundance with improved glucose and insulin tolerance when diets are also fiber rich [37, 38]. On the other hand, in studies from Westernized populations *Prevotella* dominated enterotypes (in particular *P. copri*) have been linked with autoimmune disease, new onset rheumatoid arthritis, hypertension, and diabetes [39–42]. Taken together, these data suggest that any potential health benefits from *P. copri* could be dependent on host diet background.

While overall *Bacteroides* abundance is low in NHP subjects, possibly due to the mutually exclusive dominance of *Prevotella*, monkey subjects consuming a Westernized diet did display elevated *B. uniformis and B. vulgatus* species when compared with Mediterranean diet-fed animals. In this regard, monkeys responded to translationally relevant diets in a similar pattern to humans (consumption of animal product diet increased *Bacteroides* in human subjects [12];). The duodenum, ileum, and colon of NHPs (while consuming standard monkey lab chow) are more acidic than what is observed in humans [43], which could lead to some differences in microbiota populations. *Bacteroides* have weak acidic tolerance (pH preference 6.5-6.9 [44, 45];) while *Prevotella* display slight acidic preference (pH 5.5-6.0 [46];), which could potentially explain the dominance of *Prevotella* in the gut of NHPs (human versus NHP colon pH: 6.4-7.0 (proximal to distal colon pH range) in humans compared to NHP colon pH 5.1 [41].)

We showed two distinct enterotypes in Western diet-consuming animals based upon *P. copri* abundance. The *P. copri*^LOW^ subgroup displayed increased bacterial diversity and elevated *E. siraeum* (strain: V10Sc8a). Gut *E. siraeum* abundance in NHP from either diet correlated with elevated HDL-C levels, suggesting a more metabolic healthy subgroup. Devillard et al. demonstrated novel biosynthetic activity of *E. siraeum* mediating the bioconversion of linoleic acid to form conjugated linoleic acids (CLA; mainly *cis-*9,*trans*-11-18:2 [47];). Consumption of CLA is associated with various health benefits (including elevating HDL-C [48]), further supporting the association between *E. siraeum* and HDL-C observed in our study. In a small monozygotic Korean twin study (*n*=20), *E. siraeum* negatively correlated with obesity [49], giving evidence in support of the *E. siraeum*-enriched *P. copri*^LOW^ subgroup displaying a healthier enterotype.

Our metabolic data in urine and plasma samples obtained from Western diet-fed *P. copri*^HIGH^ and *P. copri*^LOW^ subjects indicates potential early stage kidney damage in *P. copri*^HIGH^ animals. Hypertensive adolescents display elevated urinary excretion of carnitine metabolites [50] which also serve as a marker for proximal tubular damage [51]. Therefore, the observed elevated urinary carnitine, acteylcarnitine, and acyl-carnitine metabolites (but not plasma levels) may represent an attenuated capacity for reabsorption and possibly are an early mark of renal tubular damage. The uremic toxin, SDMA, inhibits nitric oxide production and is associated with coronary artery disease, type 2 diabetes mellitus, and stroke [52]. Hence, the elevated urinary SDMA observed in *P. copri*^HIGH^ animals further associates this enterotype with kidney dysfunction and may contribute to proximal tubule damage by reducing local levels of nitric oxide. Additionally, we observed elevated plasma allantoic acid concentrations in Western diet-fed *P. copri*^HIGH^ subjects. Elevated plasma allantoic acid was evident in a pre-clinical mouse model of cystic kidney disease [53]. Moreover, plasma allantoin levels were increased in both a pre-clinical rat model of kidney fibrosis [54] and in chronic kidney disease patients [55], highlighting the potential relevance of this metabolite in the assessment and/or contribution to reduced renal function (lower glomerular filtration rate). In generally healthy individuals, urine 3′sialyllactose levels correlate with chronic low-grade inflammation as measured by elevated C-reactive protein concentrations [56]. Elevated serum 3′sialyllactose served as a biomarker for mastitis development in clinically healthy dairy cows [57], giving evidence in support of 3′sialyllactose serving as a pro-inflammatory marker. The close link between chronic low-grade inflammation and the development of many metabolic diseases suggests that the elevated 3′sialyllactose observed in the urine of *P. copri*^HIGH^ may indicate a sub-clinical pro-inflammatory phenotype mediated by this gut microbiota enterotype. Elevated plasma CMPF is observed in gestational diabetes and in type 2 diabetes patients [58]. In mice, CMPF treatment (at diabetic levels) resulted in β cell dysfunction through oxidative stress demonstrating a dose-dependent causality of this metabolite to induce diabetes. However, it is important to note that fish oil administration also raises plasma CMPF levels but not to the extent observed in diabetic patients [59], suggesting key dose-dependent activity of circulating CMPF. Taken together, the identified plasma and urinary metabolomics profile support a metabolic unhealthy potential of Western diet-fed *P. copri*^HIGH^ enterotype when compared with Western diet-fed *P. copri*^LOW^ animals.

In our study, obesity (as a single variable) did not appear to be significantly associated with gut microbiome composition (Supplemental Figure S5). However, body adiposity within each diet pattern was associated with subtle shifts in the proportional abundance of several key microbiota species. Among Mediterranean diet-consuming subjects, leaner animals displayed a higher proportional abundance of *L. animalis* compared to monkeys with more adipose tissue. Several *Lactobacillus* species, including *L. animalis*, are considered healthy probiotic commensal organisms. Administration of *Lactobacillus* for 6 weeks as a probiotic supplement was shown to reduce systolic blood pressure in a controlled, randomized, double-blind trial of smokers [60]. Several preclinical studies indicate positive health benefits of *Lactobacillus* supplementation in regards to cholesterol lowering potential [61–64].

Among Western diet-fed animals, lower adiposity subjects displayed increased *B. uniformis* abundance compared to their higher adiposity counterparts. Supplementation of *B. uniformis* in obese mice fed a high-fat diet ameliorated metabolic dysfunction and reduced inflammation, indicating the elevated *B. uniformis* observed in lean Western diet-fed monkeys may be associated with a healthier phenotype [65, 66]. Heavier Western diet-fed NHPs had elevated *Ruminococcus champaneliensis* compared to their lean counterparts. *R. champanelienses* is a cellulose degrading bacterium that may supplement host metabolism by releasing digestible substrate from normally indigestible carbohydrates, potentially contributing to obesity. Within NHP on either diet, *R. champaneliensis* correlated with circulating cortisol and body fat composition, suggesting that while this species is associated with body adiposity, higher *R. champaneliensis* abundance may also modulate stress hormones in a gut-brain signaling axis.

Literature indicates that *E. rectale*, *F. prausnitzii*, *Roseburia* spp*.*, Dorea and Coprococcus metabolism produce SCFA from plant-vegetables products within the gastro-intestinal system for potential health benefits [67, 68]. We identified other *Eubacterium* species (*E. eligens*, *E. hallii*, and *Eubacterium* sp. *3_1_31*), *F. prausnitzii*, *Roseburia hominis*, multiple Dorea sp. (*D. formicigenerans*, *D. longicatena*, and *Dorea sp. AGR2135*), and several Coprococcus species (*C. catus* and *C. comes*) to be significantly higher in Mediterranean diet-fed NHP subjects than Western diet consuming animals. Our previous work demonstrated an increase in plasma bacterial modified bioactive compounds in Mediterranean diet-fed NHPs [24]. Consumption of Mediterranean diet elevated p-cresol-glucuronide, 3-indoxyl sulfate, and indole-3-propionate (IPA). IPA is the deamination product of tryptophan mediated by gut Clostridium and Lactobacillus species, which we observed elevated *Lactobacillus* sp. (*L. animalis, L. johnsonii, L. murinus,* and *L. reuteri*) and *Clostridium* sp. *SS2/1* in our Mediterranean diet-fed monkeys. Metabolomic analysis of conventional-housed and germ-free mice have indicated that germ-free mice cannot produce plasma 3-indoxyl sulfate and IPA, demonstrating the reliance on bacteria to produce these metabolites [69]. Furthermore, administration of IPA improves intestinal barrier function and decrease circulating LPS thereby reducing systemic inflammation [70]. The reduction in inflammation often observed by Mediterranean diet consumption [71–75] may result from modulation of key gut microbiota populations and the production of beneficial bacterial-derived bioactive compounds.

It is important to note several key limitations of our current study. This study only includes female NHP; sex differences may exist impacting dietary influences on the gut microbiome and metabolic outcomes in males. Baseline fecal samples were not collected during the metabolic characterization phase while the NHP were on standard monkey chow. Therefore, the data we show in this study does not accurately reflect a response to diet, but reports metabolic and microbiome patterns associated with dietary pattern consumption.

## Conclusions

In conclusion, our study demonstrated several similarities and key differences in the gut microbiome mediated by human translationally relevant diet administration. However, the key difference (stimulation of *P. copri* by Western diet administration) may represent a potential model to elucidate signaling differences observed in human Westernized populations with gut *P. copri* associations with several disease states. Furthermore, our metabolomic data indicates potential deleterious metabolite regulation in the urine and plasma of *P. copri*^HIGH^ Western diet-fed subjects, suggesting that the *P. copri*^HIGH^ enterotype in subjects eating a Westernized diet may potentiate the development of metabolic disease.

## Supplementary Information

**Additional file 1: Supplemental Table 1.** Non-human primate diets replicating human Western and Mediterranean dietary patterns. **Supplemental Figure S1.** Individual read statistics on each sample. Samples ranged from 13,694,588 to 28,756,486 reads with no significant differences in reads between dietary patterns. **Supplemental Figure S2.** Diet, not pen effects, drive microbiome populations. A. PCoA of bacterial beta diversity based on the Bray-Curtis dissimilarity; different solid color spheres indicates subjects housed in same pen. B. Shannon diversity of each subject; different solid color spheres indicates subjects housed in same pen. **Supplemental Figure S3.** Diet shifts viral and protozoa populations in the gut microbiome. A. Relative abundance of viral species in different fecal samples is visualized by bar plots. Samples are aggregated by diet cohort. Each colored box represents a viral species. The height of a color box represents the relative abundance of that organism within the sample. “Other” represents lower abundance taxa (<5%). B. Relative abundance of protozoa species in different fecal samples is visualized by bar plots. Samples are aggregated by diet cohort. Each colored box represents a protist species. The height of a color box represents the relative abundance of that organism within the sample. “Other” represents lower abundance taxa (<5%). C. Relative abundance of fungal species in different fecal samples is visualized by bar plots. Samples are aggregated by diet cohort. Each colored box represents a fungal species. The height of a color box represents the relative abundance of that organism within the sample. “Other” represents lower abundance taxa (<5%). D. A. Relative abundance of phages in different fecal samples is visualized by bar plots. Samples are aggregated by diet cohort. Each colored box represents a bacteriophage species. The height of a color box represents the relative abundance of that organism within the sample. “Other” represents lower abundance taxa (<5%). **Supplemental Figure S4.** Western diet-fed subjects with low *P. copri* abundance display elevated plasma gamma-glutamyl amino acid and phosphatidylethanolamine metabolites. **Supplemental Figure S5.** Obesity regardless of diet does not significantly regulate the gut microbiome. A. Shannon diversity. B. PCoA of bacterial beta diversity based on the Bray-Curtis dissimilarity. C. Relative abundance of bacterial species in different fecal samples is visualized by bar plots. Each bar represents a subject and each colored box a bacterial taxon. The height of a color box represents the relative abundance of that organism within the sample. “Other” represents lower abundance taxa. D. Proportional abundance of Treponema (Genus) in obese and lean NHP regardless of dietary pattern. E. Proportional abundance of *Treponema succinifaciens* in obese and lean NHP regardless of dietary pattern.

**Additional file 2.**

**Additional file 3.**

**Additional file 4.**

## Data Availability

All data generated or analyzed during this study are included in this published article [and its supplementary information files].

## Abbreviations

ANOVA: Analysis of variance
AUC: Area under the curve
BL: Body length
BW: Body weight
CLA: Conjugated linoleic acid
CMPF: 3-carboxy-4-methyl-5-propyl-2-furanpropanoate
HDL-C: High-density lipoprotein-cholesterol
IPA: Indole propionic acid
LPS: Lipopolysaccharide
NHP: Non-human primate
PCoA: Principal coordinate analysis
SCFA: Short chain fatty acid
SDMA: Symmetric dimethylarginine
TPC: Total plasma cholesterol
