# Supplementary material for: Diet, obesity, and the gut microbiome as determinants modulating metabolic outcomes in a non-human primate model
Source: Microbiome. 2021 May 5;9:100. doi: 10.1186/s40168-021-01069-y (PMC8101030; doi:10.1186/s40168-021-01069-y)

Supplemental Tables and Figures

Supplemental Table 1. Non-human primate diets replicating human Western and Mediterranean dietary patterns.

Diet Composition	Western Diet	Mediterranean Diet
**Protein** (% of calories)	16	16
**Supplemental Figures, Tables, and Legends**<br>**Carbohydrates** (% of calories)	54	54
**Fat** (% of calories)	31	31
**Saturated** (% of total fat)	36	21
**Monounsaturated** (% of total fat)	36	57
**Polyunsaturated** (% of total fat)	26	20
**n6:n3 PUFA ratio**	14.8:1	2.9:1
**Cholesterol** (mg/kcal)	0.16	0.15
**Fiber** (% of diet)	9	13
**NaCl** (g/kg)	16	6.3
**Key Ingredients**<br>(for full list see Shively *et. al.*, 2018; Shively *et. al.*, 2019)	Lard<br>Beef tallow<br>Butter<br>Corn oil<br>High fructose corn syrup<br>Salt<br>Sucrose	Fish oil<br>Olive oil<br>Fish meal<br>Dried egg whites<br>Walnut powder<br>Black and garbanzo beans<br>Fruit (banana and apple sauce)

Supplemental Figure S1. Individual read statistics on each sample. Samples ranged from 13,694,588 to 28,756,486 reads with no significant differences in reads between dietary patterns.

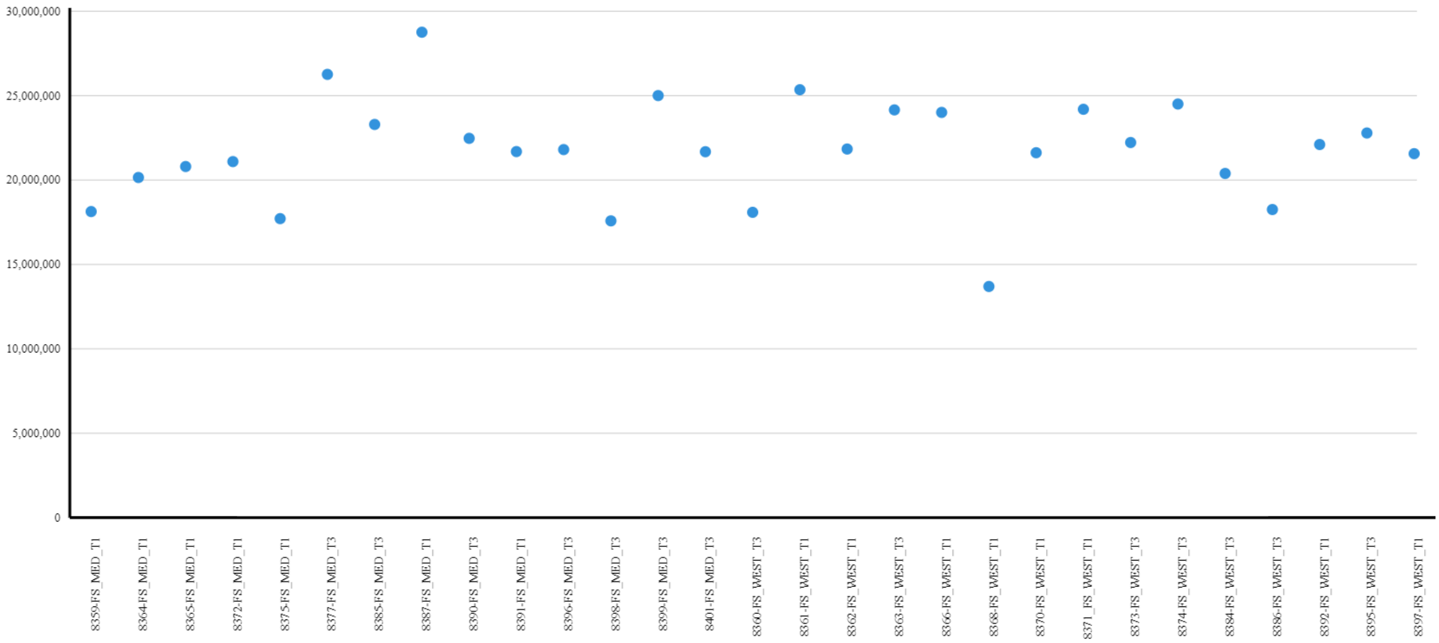

Supplemental Figure S2. Diet, not pen effects, drive microbiome populations. A. PCoA of bacterial beta diversity based on the Bray-Curtis dissimilarity; different solid color spheres indicates subjects housed in same pen. B. Shannon diversity of each subject; different solid color spheres indicates subjects housed in same pen.

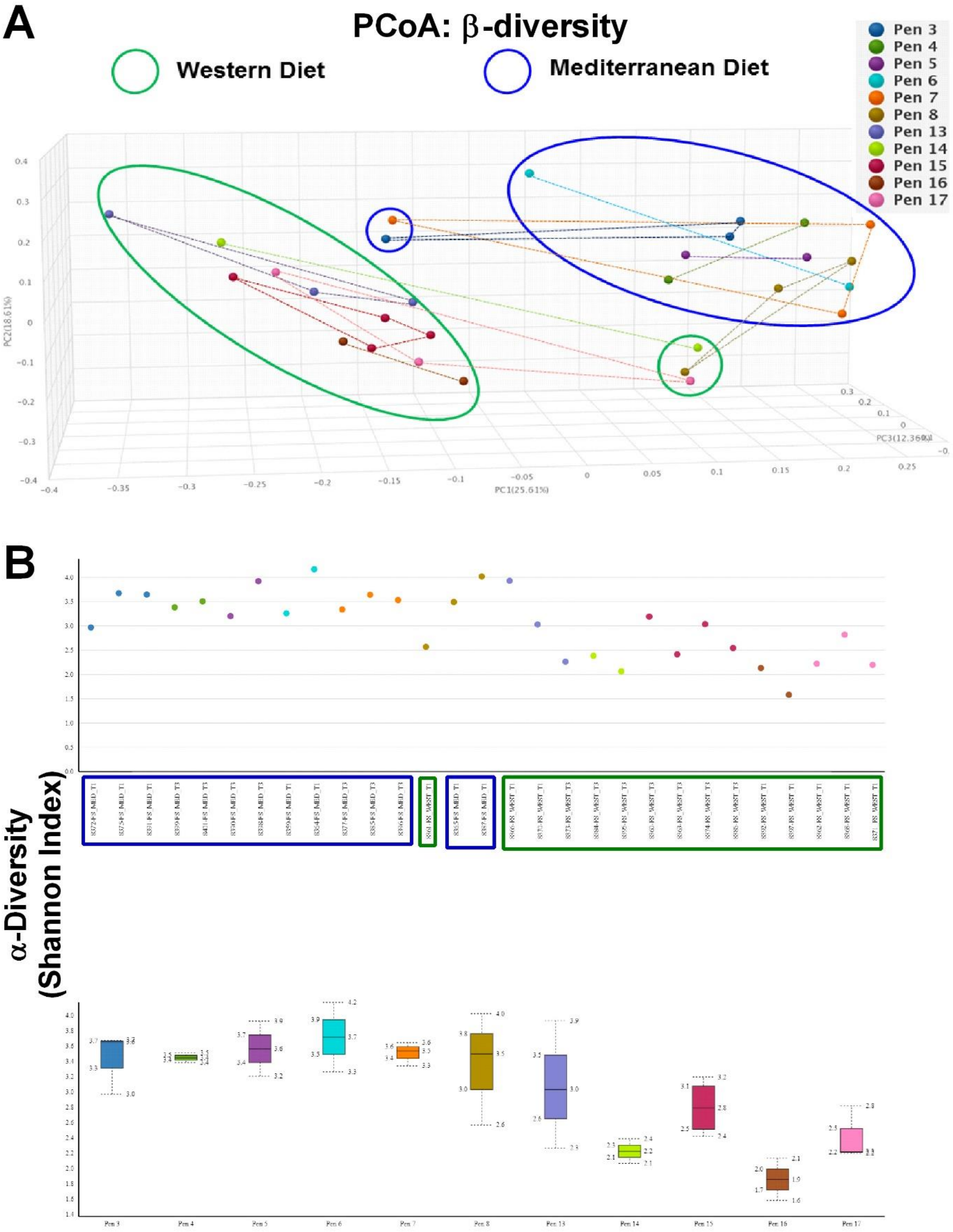

Supplemental Figure S3. Diet shifts viral and protozoa populations in the gut microbiome. A. Relative abundance of viral species in different fecal samples is visualized by bar plots. Samples are aggregated by diet cohort. Each colored box represents a viral species. The height of a color box represents the relative abundance of that organism within the sample. ‘‘Other’’ represents lower abundance taxa (<5%). B. Relative abundance of protozoa species in different fecal samples is visualized by bar plots. Samples are aggregated by diet cohort. Each colored box represents a protist species. The height of a color box represents the relative abundance of that organism within the sample. ‘‘Other’’ represents lower abundance taxa (<5%). C. Relative abundance of fungal species in different fecal samples is visualized by bar plots. Samples are aggregated by diet cohort. Each colored box represents a fungal species. The height of a color box represents the relative abundance of that organism within the sample. ‘‘Other’’ represents lower abundance taxa (<5%). D. A. Relative abundance of phages in different fecal samples is visualized by bar plots. Samples are aggregated by diet cohort. Each colored box represents a bacteriophage species. The height of a color box represents the relative abundance of that organism within the sample. ‘‘Other’’ represents lower abundance taxa (<5%)

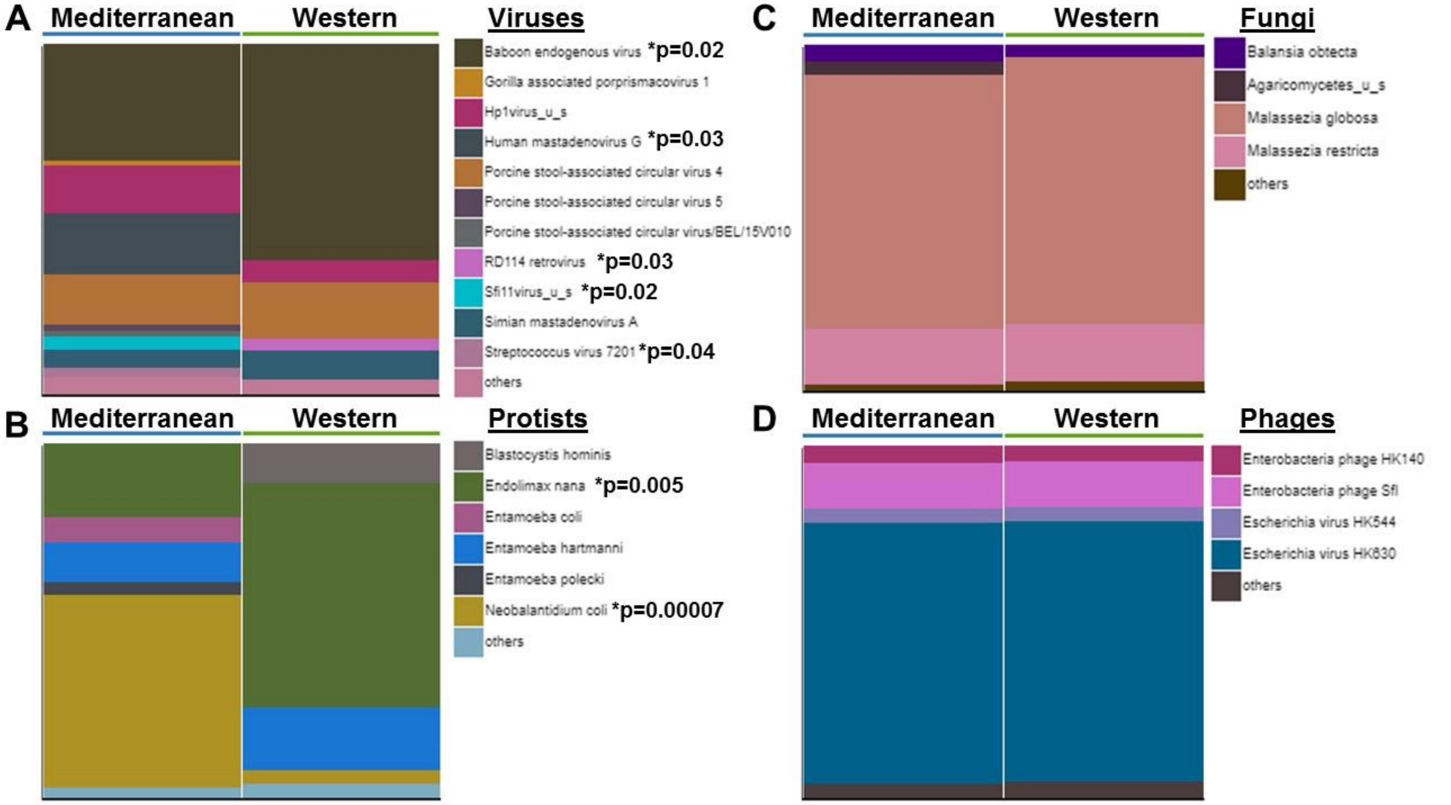

Supplemental Figure S4. Western diet-fed subjects with low *P. copri* abundance display elevated plasma gamma-glutamyl amino acid and phosphatidylethanolamine metabolites.

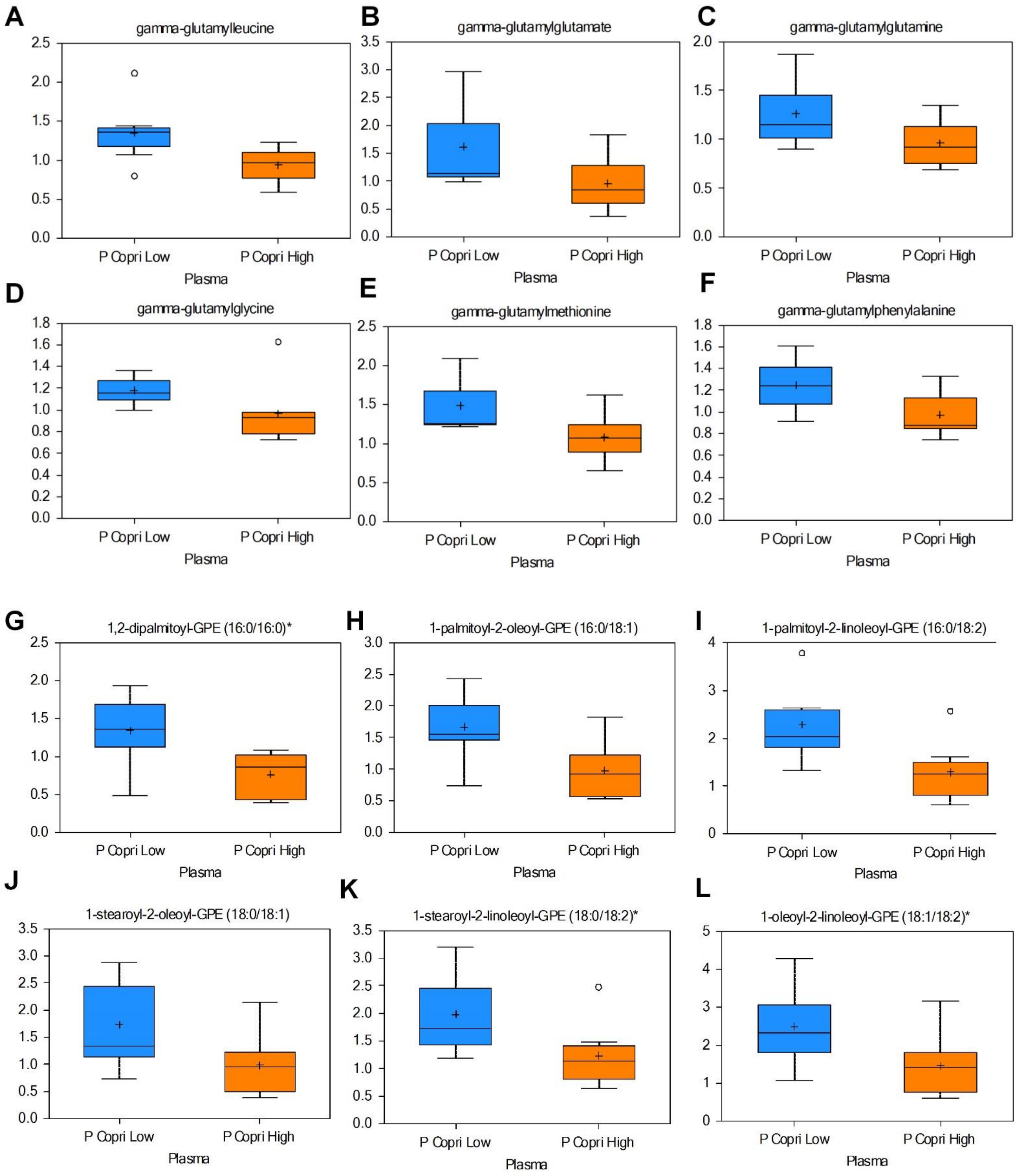

Supplemental Figure S5. Obesity regardless of diet does not significantly regulate the gut microbiome. A. Shannon diversity. B. PCoA of bacterial beta diversity based on the Bray-Curtis dissimilarity. C. Relative abundance of bacterial species in different fecal samples is visualized by bar plots. Each bar represents a subject and each colored box a bacterial taxon. The height of a color box represents the relative abundance of that organism within the sample. ‘‘Other’’ represents lower abundance taxa. D. Proportional abundance of Treponema (Genus) in obese and lean NHP regardless of dietary pattern. E. Proportional abundance of *Treponema succinifaciens* in obese and lean NHP regardless of dietary pattern.

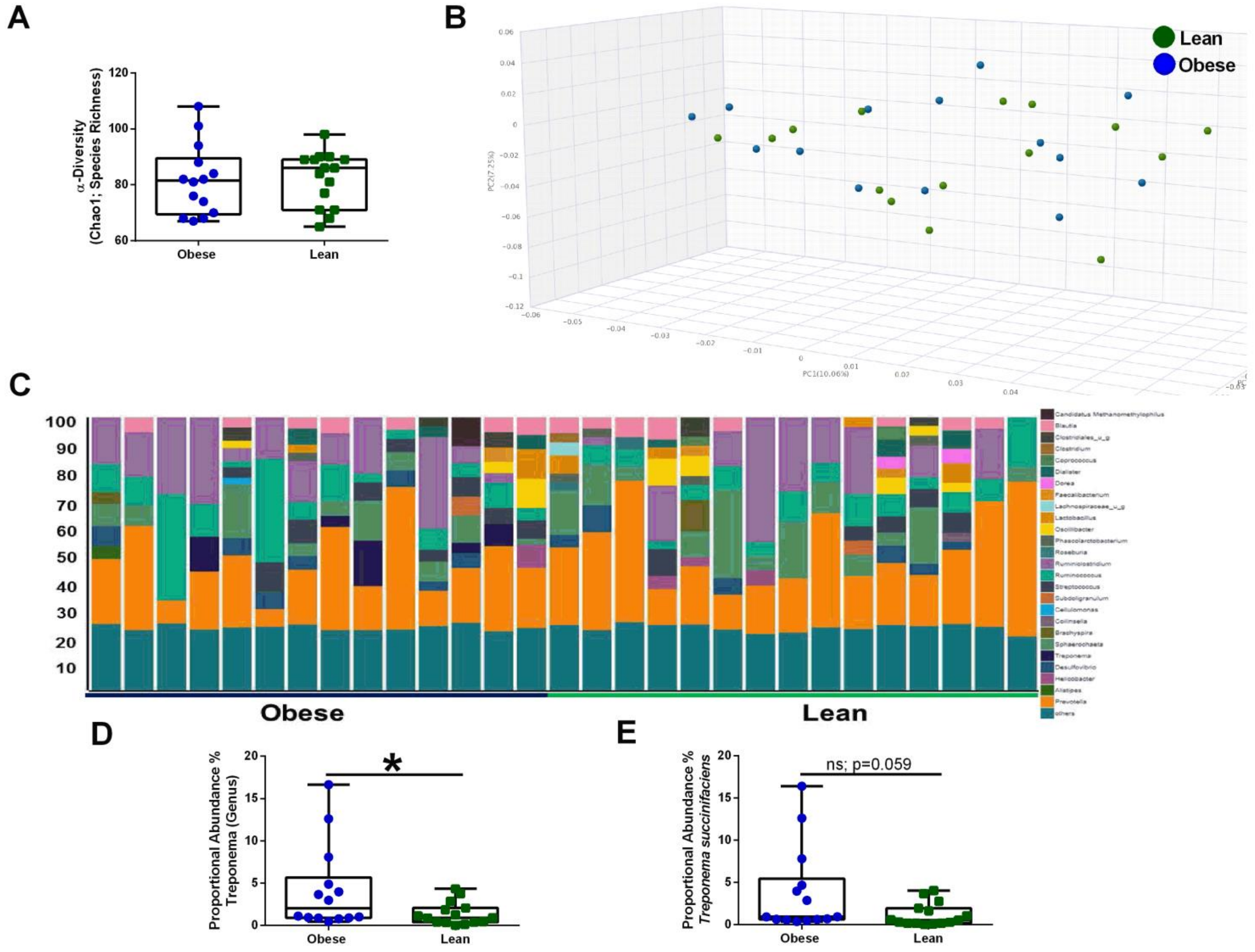

Supplement: Supplementary file 2 — Additional file 1: Supplemental Table 1. Non-human primate diets replicating human Western and Mediterranean dietary patterns. Supplemental Figure S1. Individual read statistics on each sample. Samples ranged from 13,694,588 to 28,756,486 reads with no significant differences in reads between dietary patterns. Supplemental Figure S2. Diet, not pen effects, drive microbiome populations. A. PCoA of bacterial beta diversity based on the Bray-Curtis dissimilarity; different solid color spheres indicates subjects housed in same pen. B. Shannon diversity of each subject; different solid color spheres indicates subjects housed in same pen. Supplemental Figure S3. Diet shifts viral and protozoa populations in the gut microbiome. A. Relative abundance of viral species in different fecal samples is visualized by bar plots. Samples are aggregated by diet cohort. Each colored box represents a viral species. The height of a color box represents the relative abundance of that organism within the sample. “Other” represents lower abundance taxa (<5%). B. Relative abundance of protozoa species in different fecal samples is visualized by bar plots. Samples are aggregated by diet cohort. Each colored box represents a protist species. The height of a color box represents the relative abundance of that organism within the sample. “Other” represents lower abundance taxa (<5%). C. Relative abundance of fungal species in different fecal samples is visualized by bar plots. Samples are aggregated by diet cohort. Each colored box represents a fungal species. The height of a color box represents the relative abundance of that organism within the sample. “Other” represents lower abundance taxa (<5%). D. A. Relative abundance of phages in different fecal samples is visualized by bar plots. Samples are aggregated by diet cohort. Each colored box represents a bacteriophage species. The height of a color box represents the relative abundance of that organism within the sample. “Other” represents lo [file 40168_2021_1069_MOESM2_ESM.pdf]
